# 

**Published:** 1976

**Authors:** 


					VV/ n
-11 3 (V
BRISTOL
MEDICO-
CHIRURGICAL
JOURNAL
JULV/OCTOBER, 1976
Vol. 91 (iii/iv) IMo. 339/340

				

